# Audit on the monitoring of metabolic side effects of antipsychotics in acute inpatient psychiatric units at Fieldhead Hospital

**DOI:** 10.1192/bjo.2021.200

**Published:** 2021-06-18

**Authors:** Stephanie Vel En Tial, Steve Curran, Adebayo Ikuyajesin

**Affiliations:** Fieldhead Hospital, South West Yorkshire Partnership Trust

## Abstract

**Aims:**

The current audit aims to assess the compliance with Prescribing Observatory for Mental Health (POMH-UK) guidance on monitoring of metabolic side effects of patients prescribed antipsychotics. Compliance was monitored to ensure that all patients prescribed continuing antipsychotics have their body mass index (BMI), blood pressure, blood glucose and lipids checked within the expected time limits of minimum once per year.

**Background:**

Patients diagnosed with Schizophrenia rank amongst the worst of chronic medical illnesses in terms of quality of life. This may in part be due to the use of long term antipsychotic medications, in particular the use of atypical antipsychotics which have been increasingly associated with metabolic side effects including hypertension, weight gain, glucose intolerance and dyslipidaemia. These side effects are related to the development of both diabetes mellitus and cardiovascular disease and can lead to increased mortality and morbidity, affecting compliance and engagement to healthcare services. Despite the availability of clinical guidelines, monitoring and screening of metabolic side effects in patients prescribed antipsychotics continues to be suboptimal.

**Method:**

The audit involved a review of electronic records relating to physical health monitoring of patients at two acute inpatient units from January-March 2019. Demographic and clinical variables were collected which included ethnicity, diagnostic grouping as well as current medications. Data were collected on evidence of screening for hypertension, BMI, blood glucose and lipids. Descriptive statistics were applied to study the clinical features of the sample and examine whether performance met clinical practice standard.

**Result:**

The audit overall demonstrated partial compliance with POMH-UK guidelines with a total of 31 patients admitted on long term antipsychotics. Of these patients, 86% were prescribed atypical antipsychotics with 14% prescribed typical antipsychotics. Screening only occurred in 68% of patients for lipid profile with only 71% for BMI and 74% for blood glucose. Blood pressure had the highest compliance rate of 87% of patients being screened.

**Conclusion:**

Early identification and monitoring of complications from metabolic syndrome may decrease the risk of more serious health outcomes and improve patients’ quality of life. However in clinical practice, standards are not always met in accordance with best practice recommendations. Requirement of a tailored guideline for physical health monitoring with weekly planned interventions as well as adequate training and awareness of healthcare staff is imperative to drive improvement and increase adherence rates.

